# Transdiagnostic individualized clinically-based risk calculator for the automatic detection of individuals at-risk and the prediction of psychosis: external replication in 2,430,333 US patients

**DOI:** 10.1038/s41398-020-01032-9

**Published:** 2020-10-29

**Authors:** Dominic Oliver, Chiew Meng Johnny Wong, Martin Bøg, Linus Jönsson, Bruce J. Kinon, Allan Wehnert, Kristian Tore Jørgensen, Jessica Irving, Daniel Stahl, Philip McGuire, Lars Lau Raket, Paolo Fusar-Poli

**Affiliations:** 1 Early Psychosis: Interventions and Clinical detection (EPIC) Lab, Department of Psychosis Studies, Institute of Psychiatry, Psychology and Neuroscience, King’s College London, London, SE5 8AF UK; 2 Lundbeck Singapore Pte, Ltd., Singapore, 307591 Singapore; 3 H. Lundbeck A/S, Valby, Denmark; 4 Karolinska Institutet, Stockholm, Sweden; 5 Lundbeck Pharmaceuticals LLC, Deerfield, IL 60015 USA; 6 Department of Biostatistics, Institute of Psychiatry, Psychology and Neuroscience, King’s College London, London, SE5 8AF UK; 7 OASIS Service, South London and the Maudsley NHS National Health Service Foundation Trust, London, SE5 8AZ UK; 8 Department of Psychosis Studies, Institute of Psychiatry, Psychology and Neuroscience, King’s College London, London, SE5 8AF UK; 9 Clinical Memory Research Unit, Lund University, Lund, Sweden; 10 Department of Brain and Behavioural Sciences, University of Pavia, 27100 Pavia, Italy

**Keywords:** Schizophrenia, Prognostic markers

## Abstract

The real-world impact of psychosis prevention is reliant on effective strategies for identifying individuals at risk. A transdiagnostic, individualized, clinically-based risk calculator to improve this has been developed and externally validated twice in two different UK healthcare trusts with convincing results. The prognostic performance of this risk calculator outside the UK is unknown. All individuals who accessed primary or secondary health care services belonging to the IBM^®^ MarketScan^®^ Commercial Database between January 2015 and December 2017, and received a first ICD-10 index diagnosis of nonorganic/nonpsychotic mental disorder, were included. According to the risk calculator, age, gender, ethnicity, age-by-gender, and ICD-10 cluster diagnosis at index date were used to predict development of any ICD-10 nonorganic psychotic disorder. Because patient-level ethnicity data were not available city-level ethnicity proportions were used as proxy. The study included 2,430,333 patients with a mean follow-up of 15.36 months and cumulative incidence of psychosis at two years of 1.43%. There were profound differences compared to the original development UK database in terms of case-mix, psychosis incidence, distribution of baseline predictors (ICD-10 cluster diagnoses), availability of patient-level ethnicity data, follow-up time and availability of specialized clinical services for at-risk individuals. Despite these important differences, the model retained accuracy significantly above chance (Harrell’s C = 0.676, 95% CI: 0.672–0.679). To date, this is the largest international external replication of an individualized prognostic model in the field of psychiatry. This risk calculator is transportable on an international scale to improve the automatic detection of individuals at risk of psychosis.

## Introduction

Under standard care, clinical outcomes in psychosis are suboptimal; prevention and early intervention are essential to improve outcomes of this disorder^1^. Primary indicated prevention of psychosis revolves around the ability to detect, assess and care for individuals at risk of psychosis. The Clinical High Risk state for Psychosis (CHR-P)^2^ includes individuals who present with attenuated psychotic symptoms, impaired functioning^3^ and help-seeking behavior. Twenty percent of these individuals develop a psychotic disorder within two years^4^. Primary indicated prevention of psychosis through specialized CHR-P clinical services^5^ is uniquely positioned to alter the course of the disorder and improve outcomes^1^.

The impact of the CHR-P approach is contingent on effective identification of individuals at risk of developing psychosis. Because of complex interactions between help-seeking behaviors, recruitment strategies and referral pathways^6^, detection of at-risk individuals is currently inefficient: only 5%^7^–12%^8^ of first-episode cases are identified by specialized or youth mental health CHR-P services. Moreover, these services are only available to a limited number of individuals, with only 48 services mapped worldwide^9^. To overcome these problems, a transdiagnostic, individualized, clinically-based risk calculator has been developed in the South London and Maudsley (SLaM) NHS Trust boroughs of Lambeth and Southwark (*n* = 33,820)^7^. This prognostic model uses core predictors that were selected on *a priori* meta-analytical knowledge^10^ (age, gender, ethnicity, primary index diagnosis and age*gender interaction), that are routinely collected in clinical care, to forecast individual level of psychosis risk up to six years. This model leverages electronic health record (EHR) data, therefore allowing for the automatic detection of at-risk individuals. This prognostic model has shown adequate performance in a first external validation in the SLaM boroughs of Lewisham and Croydon (*n* = 54,716, Harrell’s C = 0.79)^7^ and in a second external validation in the Camden and Islington NHS Foundation Trust (C&I; *n* = 13,702, Harrell’s C = 0.73)^11^, with Harrell’s C demonstrating the probability that a randomly selected patient who experienced an event will have a higher score than a patient who did not. This prognostic model is also currently being piloted for real-world implementation in clinical routine in the UK^12^.

Despite these promising results, it is not yet clear whether this prognostic model is transportable to international healthcare settings. External validation studies are scarce in psychiatry, undermining the translational impact of research discoveries. This study aims to investigate the international external validity of the original transdiagnostic, clinically-based, individualized risk calculator using large scale EHRs from the US.

## Materials and methods

### Design

Retrospective cohort study using Electronic Health Records (EHRs) conducted according to the *REporting of studies Conducted using Observational Routinely-collected health Data (RECORD)* statement^13^ (see checklist reported in Table S1).

### Data source

The IBM^®^ MarketScan^®^ Commercial Database (hereafter Commercial) contains data from approximately 65 million people from multiple geographically dispersed US states, who are covered by employer-sponsored health insurance plans. This data includes all medical and pharmaceutical claims for these individuals and their dependents (Methods S1). It provides contemporaneous and ‘real-world’ data on both routine primary and secondary mental healthcare.

### Study population

All patients accessing primary or secondary healthcare between 1 January 2015 and 31 December 2017 who received an ICD-10 primary index diagnosis of a nonorganic and nonpsychotic mental disorder (Methods S2). To ensure correct diagnosis classification, a lookback period of six months was applied to each patient (Methods S3).

### Follow-up

Follow-up started at the time of the ICD-10 index diagnosis and ended when a transition to psychosis was recorded, or when the patient dropped out of the EHR (as documented by the last entry on Commercial).

### Model specifications

The original transdiagnostic, clinically-based, individualized risk calculator was developed using a retrospective cohort study leveraging EHRs of the SLaM boroughs of Lambeth and Southwark, firstly validated in the SLaM boroughs of Croydon and Lewisham^7^ and secondly validated in C&I^11^ in the UK. In summary, a Cox model was used to predict the hazard ratio of developing any psychotic disorder over time (see Methods S2 for definition) as primary outcome of interest. The predictors included age (at the time of the index diagnosis), gender, age*gender, self-assigned ethnicity, and cluster index diagnosis (ICD-10 diagnostic spectra: acute and transient psychotic disorders (ATPD), substance use disorders, bipolar mood disorders, nonbipolar mood disorders, anxiety disorders, personality disorders, developmental disorders, childhood/adolescence onset disorders, physiological syndromes, mental retardation). Self-assigned ethnicity and index diagnoses were operationalized as indicated in Tables S2 and S3. A weighted sum of covariates with the model weights from the Cox model resulted in the Prognostic Index (PI). From this, the risk of the individual developing a psychotic disorder within a time period (between one and six years) could be calculated^14^.

Since this model was originally developed on a retrospective cohort^7^, it excluded cases with an onset of psychosis within the first three months to minimize the short-term diagnostic instability of baseline ICD-10 index diagnoses. However, during the subsequent implementation study^12,15^ an updated version of the model was adapted for prospective use (i.e., not excluding transitions occurring in the first three months), demonstrating similar prognostic performance (Table S4). Furthermore, a lookback period was additionally used in this study (see Methods S3), to minimize the risk of misclassification of index diagnosis date. The specifications of the present model are fully detailed in Table S5.

A main difference compared to the SLaM dataset was that there were no patient-level ethnicity data in Commercial. To mitigate this issue, aggregate ethnicity coefficients were generated for patients who had Metropolitan Statistical Area (MSA) and state-level ethnicity data using Integrated Public Use Microdata Series (IPUMS) census data (www.ipums.org). The geographical information from IPUMS were matched with the geographical data available for each patient in the study population from Commercial, assigning each patient with a vector of ethnic weights for each level of the ethnicity predictor. For example, if a patient were matched for New York (NY) state and Ithaca, NY MSA and was diagnosed in 2016, the proportions of White individuals in the MSA in the year of index diagnosis was 0.82, Black individuals was 0.03, Asian individuals was 0.10, Mixed individuals was 0.03 and Other was 0.01. For comparability purposes we also reported the performance of the original model^7^ (i) without ethnicity as a predictor and (ii) with computed aggregate ethnicity using census data^16^ (Table S6).

### Statistical analysis

Model external validation followed the guidelines of Royston and Altman^17^, Steyerberg et al.^18^, and the Transparent Reporting of a multivariable prediction model for Individual Prognosis Or Diagnosis (TRIPOD)^19^. The study protocol is uploaded in the Research Registry database (www.researchregistry.com, researchregistry5130).

For a general overview of prognostic modeling methods, including external validation procedures, see our recent review^20^. To interpret the performance of a risk model in the context of external validation, it is essential to first quantify the similarities between development and validation samples^21^. External validity only assesses model transportability if validation samples have a different case-mix, with the greater the difference in the case-mixes, the greater the possibility of generalizing to other populations. Thus, we investigated the extent to which the SLaM and Commercial datasets comprised patients with sets of prognostically relevant predictors in common, comparable time to event outcomes with roughly similar follow-up times, and the same clinical condition observed in similar settings^22^.

As a first step, we described the Commercial patient population, including the configuration of clinical services and compared with SLaM. Baseline clinical and sociodemographic characteristics of the sample (including missing data) were described by means and SDs for continuous variables, and absolute and relative frequencies for categorical variables^22^.

In a second step, we visually compared the two Kaplan–Meier failure functions, showing the number of patients developing a psychotic disorder, as well as those still at risk, over time. The overall cumulative risk of psychosis onset in Commercial was visualized with the Kaplan–Meier failure function (1—survival)^23^ and Greenwood 95% confidence intervals (CIs)^24^. Curves that vary noticeably may indicate systematic differences within the study populations^22^.

In a third step, we reported the spread (SD) and mean of the PI in the two datasets. An increased (or decreased) variability of the PI would indicate more (or less) heterogeneity of case-mix between the two datasets, and therefore, of their overarching target populations^21^. Differences in the mean PI indicate differences in overall (predicted) outcome frequency, reflecting case-mix severity between the two datasets (and revealing the model’s calibration-in-the-large in the Commercial database)^21^. Continuous variables were tested with independent *t*-tests.

We then performed the formal external validation, assessing the prognostic accuracy of the model in the Commercial database^22^. Accordingly, the regression coefficients obtained from our model developed in SLaM (see Table S6) were applied to each case in the external Commercial database, to generate the PI in the Commercial database. In the case of ethnicity, the aggregate ethnic weights were multiplied by their respective regression coefficients to provide an aggregate coefficient for that patient. The sum of an individual’s regression coefficients resulted in an individualized PI. The greater the PI, the higher the risk of the individual developing a psychotic disorder.

Since we were interested in discrimination, the primary outcome measure for this study was the external model performance (accurate predictions discriminate between those with and those without the outcome)^18^, defined with the Harrell’s C-index^17^. Harrell’s C is a recommended measure for external validation of Cox models according to established guidelines^17^. Harrell’s C is the probability that for a random pair of “case” and “control,” the predicted risk of an event (PI) is higher for the “case”^25^. In addition, we estimated the overall model performance^18^ using the Brier score (average mean squared difference between predicted probabilities and actual outcomes, which also captures calibration and discrimination aspects)^18^. Calibration (agreement between observed outcomes and predictions)^18^ was assessed using the regression slope of the PI^17,18^.

As a further exploratory step, we updated the model using the regression slope on the PI as a shrinkage factor for recalibration, in line with the Royston et al. guidelines^22^.

All analyses were conducted in R version 3.3.2^26^. using the survival package, and significance was set to *P* < .05.

## Results

### Commercial sample characteristics

**Fig. 1:**
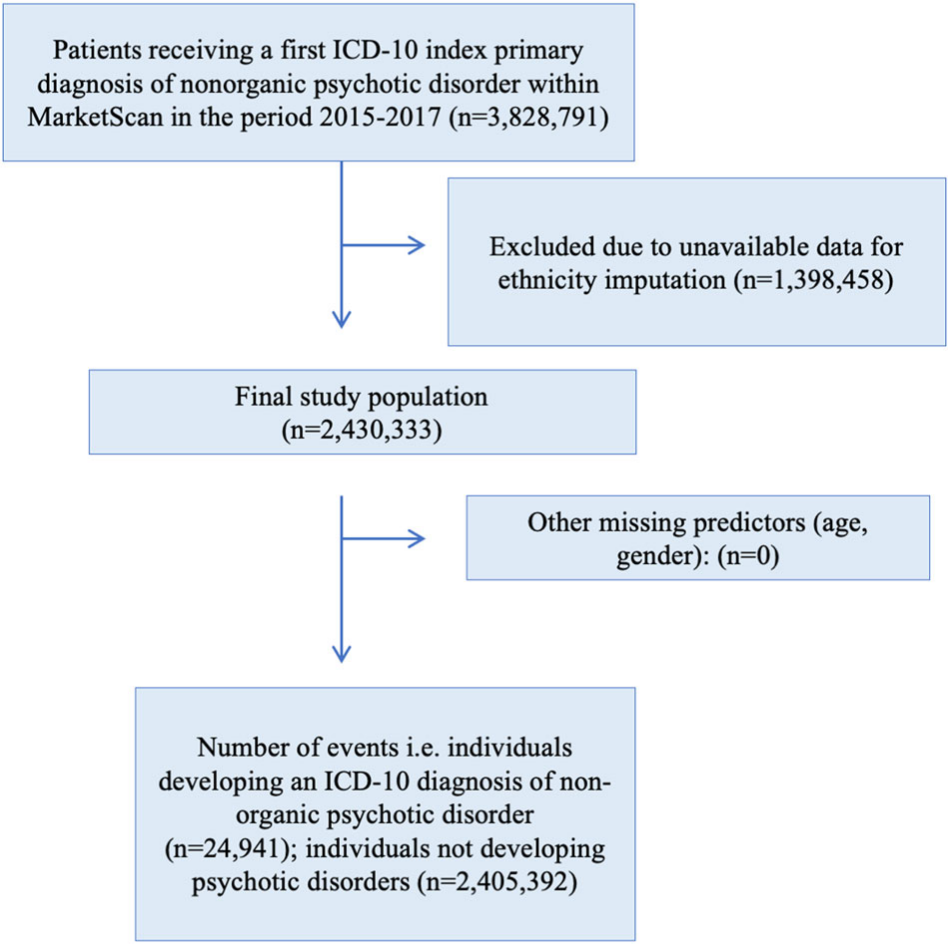
Flowchart of the study population. 3,828,791 patients received a first ICD-10 index primary diagnosis of a nonorganic psychotic disorder. 1,398,458 patients were excluded as there was not sufficient data available to impute aggregate ethnicity coefficients. This provided a final study population of 2,430,333, which included 24,941 individuals who developed an ICD-10 diagnosis of a non-organic psychotic disorder.

A total of 3,828,791 patients accessing primary or secondary healthcare between January 2015 and December 2017 received an ICD-10 primary index diagnosis of a nonorganic and nonpsychotic mental disorder. 2,430,333 (63.5%) of these individuals could be matched with ethnicity data, and were included in the analysis, as detailed in the study flow-diagram (Fig. 1). Patients accessing Commercial and included in this study had an average age of 34.2 years (95% CI: 34.19–34.23), 59% were female, and White ethnicity was particularly common in patients’ MSAs (79%). The most frequent index diagnosis was anxiety disorders (45%). Full sociodemographic information is provided in Table 1.

**Table 1:** Sociodemographic characteristics of the commercial dataset compared with the SLaM dataset.

	Commercial (external validation database) (*n* = 2,430,333) Mean (SD)	SLaM (original development database) (*n* = 34,209) Mean (SD)
Age, years	34.2 (16.88)	34.43 (18.89)
Ethnicity(a)		No. (%)
Black	0.12 (0.10)	7,055 (22.19)
White	0.79 (0.11)	18,768 (59.03)
Asian	0.04 (0.04)	1,149 (3.61)
Mixed	0.03 (0.01)	1,319 (4.15)
Other	0.02 (0.03)	3,502 (11.02)
Sex	No. (%)	No. (%)
Male	995,262 (40.95)	17,511 (51.20)
Female	1,435,071 (59.05)	16,688 (48.80)
Index diagnosis	No. (%)	No. (%)
CHR-P	-	314 (0.92)
Acute and transient psychotic disorders	1,316 (0.05)	747 (2.18)
Substance use disorders	153,401 (6.31)	7,187 (21.01)
Bipolar mood disorders	64,623 (2.66)	980 (2.86)
Nonbipolar mood disorders	543,854 (22.38)	6,364 (18.60)
Anxiety disorders	1,092,893 (44.97)	8,279 (24.20)
Personality disorders	11,572 (0.48)	1,297 (3.79)
Developmental disorders	74,072 (3.05)	1,413 (4.13)
Childhood/adolescence onset disorders	418,316 (17.21)	4,201 (12.28)
Physiological syndromes	68,476 (2.82)	2,560 (7.48)
Mental retardation	1,810 (0.07)	867 (2.53)

(a) Ethnicity data in Commercial were imputed so they are not directly comparable with SLaM. The means and SDs presented here represent the average proportion of ethnicities in patients’ Metropolitan Statistical Area (MSA).

### Differences between the commercial and SLaM databases

#### Sociodemographic and service configuration differences

The most important difference is that while the SLaM database contains data on individuals accessing publicly funded secondary mental healthcare, Commercial is limited to individuals covered by employer-sponsored health insurance plans. Compared to the full population, incidence of psychosis may be rarer in those covered by private insurance such as in the Commercial dataset. Similar to the C&I Trust that was the basis of the second external replication study, Commercial did not include CHR-P services; therefore, there were no CHR-P diagnoses. Additional differences are that Commercial data incorporates both primary and secondary healthcare, compared to solely secondary healthcare in SLaM and C&I, as well as the aggregation of ethnicity data as discussed in “Methods” section. The average patient’s age in the Commercial was 0.2 years lower than in SLaM (*p* = 0.03). Compared with SLaM, there was a lower incidence of ATPD, substance use disorders, bipolar mood disorders, personality disorders, developmental disorders, physiological syndromes and mental retardation in the Commercial dataset. Conversely, there were higher rates of nonbipolar mood disorders, anxiety disorders and childhood/adolescence onset disorders. Finally, there were fewer males in Commercial than in SLaM (Table 1).

#### Cumulative risk of psychosis in commercial compared with the SLaM derivation dataset

**Fig. 2:**
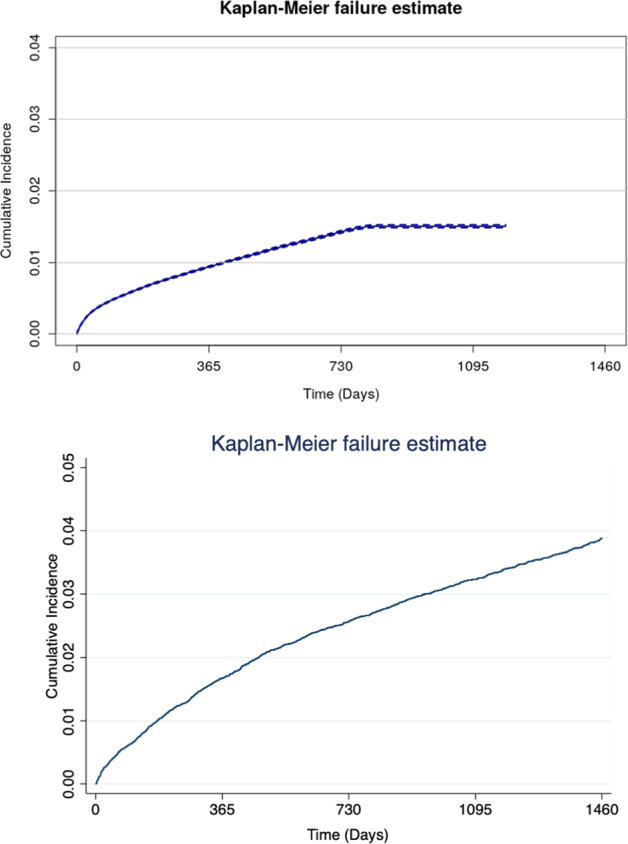
Cumulative incidence for the risk of psychotic disorders in Commercial Database and SLaM derivation database. Upper part of the figure: cumulative incidence (Kaplan–Meier failure function) for risk of development of psychotic disorders in the Commercial Database. There were a total of 24,941 events (transition to psychosis): 19,687 in the first 365 days, 4,851 in the interval 366–730 days, 403 in the interval 731–819 days. The last event was observed at 819 days, when 360,396 individuals were still at risk. The cumulative incidence of psychosis was: 0.94 (95% CI: 0.93–0.95) at one year and 1.43 (95% CI: 1.41–1.45) at two years. Lower part of the figure: cumulative incidence (Kaplan–Meier failure function) for risk of development of psychotic disorders in the SLaM derivation database, truncated at 1,460 days for visual comparability. Cumulative incidence of psychosis: 1.67 (95% CI: 1.61–1.89, 30,102 individuals still at risk) at one year, 2.57 (95% CI: 2.40–2.75, 26,337 individuals still at risk) at two years.

The average follow-up time in Commercial was 460.89 days (SD = 280.04) compared with 1580.64 days (SD = 927.72) in SLaM. There were 24,941 (1.03% of the sample size) events (transition to psychosis) in Commercial compared with 1,273 (3.72% of the sample size) in SLaM. The average time to transition to psychosis in those who transitioned was 199.77 days (SD = 204.48) in Commercial compared to 664.03 days (SD = 621.04) in SLaM. The two-year cumulative risk of psychosis in the Commercial was 1.43% (95% CI: 1.41–1.45%, with the last transition being observed at 819 days), compared to 2.57% (95% CI: 2.40%–2.75%, with the last transition being observed at 3,246 days) in SLaM. The cumulative incidences curves (Kaplan–Meier) from the Commercial and SLaM datasets are compared in Fig. 2. Mean values of the PI within the Commercial and SLaM databases were −1.51 and −1.18, respectively (*P* < .001). SD of the PI in the Commercial and SLaM databases were 0.70 and 0.94, respectively (*P* < .001).

### External validation in the commercial database

The comparative model performance in the SLaM dataset using aggregate ethnicity data was 0.761 (Table S5). In the Commercial dataset, the model predicted significantly better than chance, with a Harrell’s C of 0.676 (95% CI: 0.672–0.679, Harrell’s C in SLaM = 0.79). The two-year Brier score was 0.013 (two-year Brier score in SLaM = 0.012). The model did not show major calibration issues, with a regression slope close to 1: 0.93, 95% CI: 0.91–0.94 (*P* < .001).

Updating the model optimized calibration (regression slope = 1) but conferred no substantial improvement in model performance (full model specifications are appended in Table S6).

## Discussion

This is the largest ever replication study of a risk prediction model in psychiatry. The study demonstrates that the transdiagnostic, individualized risk calculator was able to detect individuals at risk of psychosis in an international setting with a prognostic discriminative performance that was significantly above chance.

To our knowledge, this is the largest ever external replication study of a risk calculator not only in early psychosis but also in clinical psychiatry. Importantly, this study included 24,941 events (transitions to psychosis) which are over one hundred times the minimum recommended amount of 100 events required to produce accurate estimates of external prognostic accuracy^20,27^. The previous largest external validation study of this kind was our first external replication of this calculator conducted in SLaM (*n* = 33,820)^7^, followed by a validation study of a calculator that predicts major depressive disorder (*n* = 29,621)^28^ and by another calculator that predicts risk of violent crime in patients with severe mental illness (*n* = 16,387)^29^, all smaller than our sample size of 2,40,333. This is a substantial achievement given that prognostic modeling in psychiatry is affected by a severe scarcity of replication efforts^30^, to the point that replication has become equally as—or even more—important than discovery^31^. A systematic review and meta-analysis of clinical prediction models for predicting the onset of psychosis in CHR-P people uncovered 91 studies, none of which performed a true external validation of an existing model^32^. This is the only transdiagnostic clinical prediction model to be externally validated in three different populations (Lewisham & Croydon SLaM NHS Trust, C&I and now Commercial); another risk prediction model for use in CHR-P patients has also received three independent validations^33–35^. A full list of individualized risk prediction models that have been externally replicated in the field of early psychosis is detailed in Table 2.

**Table 2:** Individualized clinical prediction models that have been externally validated for early psychosis.

Author		Year	Targets	Population	Derivation sample size (Location)	Performance	Validation sample size (Location)	Performance	Data	
									CLIN	NPSY
Fusar-Poli^55^		2016	Detection	CHR-P	321 (UK)	Harrell’s C = 0.66	389 (UK)	Harrell’s C = 0.66	Y	
Fusar-Poli^7^		2017	Detection	CHR-P	33,820 (UK)	Harrell’s C = 0.80	54,716 (UK) 13,702 (UK)^11^ 2,430,333 (USA)	Harrell’s C = 0.79 Harrell’s C = 0.73 Harrell’s C = 0.68	Y	
	Refined: Natural language processing^59^				28,297 (UK)	Harrell’s C = 0.86	63,854 (UK)	Harrell’s C = 0.85	Y	
	Refined: Non-linear modelling of age^49^				33,820 (UK)	Harrell’s C = 0.81	54,716 (UK)	Harrell’s C = 0.81	Y	
Cannon^42^		2016	Prognosis (Transition)	CHR-P	596 (USA)	Harrell’s C = 0.71	176 (USA)^33^ 68 (USA)^34^ 199 (China)^35^	AUC = 0.79 AUC = 0.71 AUC = 0.63	Y	Y
Zhang^43^		2019	Prognosis (Transition)	CHR-P	349 (China)	AUC = 0.74	100 (China) 68 (USA)^34^	AUC = 0.80 AUC = 0.65	Y	Y
Koutsouleris^56^		2016	Prognosis (Functioning)	FEP	334 (Europe, Israel)	BAC = 0.75	108 (Europe, Israel)	BAC = 0.72	Y	
Leighton^57^		2019	Prognosis (Functioning)	FEP	83 (UK)	NR	79 (UK)	AUC = 0.88	Y	
Leighton^58^		2019	Prognosis (Remission, Recovery, Quality of life)	FEP	Remission: 673 (UK) Social recovery: 829 (UK) Vocational recovery: 807 (UK) Quality of life: 729 (UK)	Remission: AUC = 0.70 Social recovery: AUC = 0.73 Vocational recovery: AUC = 0.74 Quality of life: AUC = 0.70	Remission: 131 (UK) Vocational recovery: 142 (UK) Quality of life: 47 (UK)	Remission: AUC = 0.68 Vocational recovery: AUC = 0.87 Quality of life: AUC = 0.68	Y	
							Remission: 338 (Denmark) Social recovery: 518 (Denmark) Vocational recovery: 553 (Denmark) Quality of life: 226 (Denmark)	Remission: AUC = 0.62 Social recovery: AUC = 0.57 Vocational recovery: AUC = 0.66 Quality of life: AUC = 0.56	Y	

This table presents key features of the target populations, discrimination/prognostic performance and type of data used in externally validated individualized clinical prediction models for early psychosis. Population: CHR-P clinical high risk for psychosis, FEP first-episode psychosis; Performance: AUC area under the curve, BAC balanced accuracy, NR not reported; Data: CLIN clinical data, NPSY neuropsychological data, Y yes.

The additional strength of this study is that it provides further empirical support for the use of EHRs in the context of precision psychiatry. Transporting risk prediction models across different EHRs representing heterogeneous clinical settings is complex because they reflect underlying differences in the patient population. A first empirical challenge is the availability of predictors and outcomes. The vast majority of predictors were available in the Commercial database, with the exception of ethnicity; patient-level ethnicity variables were computed to compensate for this. There was also a shorter follow-up time in Commercial compared to SLaM, as ICD-10 was only integrated into United States healthcare on 1 October 2015. Use of ICD-9 diagnoses was considered to extend follow-up but converting diagnostic clusters to ICD-9 proved inexact and therefore inappropriate. A second challenge is to quantify the differences between development and validation databases to interpret the performance of a risk model in the context of external validation^21^. For example, compared with SLaM, where the model was developed, there were apparent differences in sociodemographic characteristics in Commercial (fewer males and fewer patients of Black ethnicity and different frequency of ICD diagnoses, reflected by smaller spread of the PI) and time to event (shorter). Furthermore, similar to our second replication in C&I^11^, there were no CHR-P services in Commercial and, therefore, no CHR-P designations. However, as ATPD diagnoses are typically not made in CHR-P or early intervention services^36^, the number of ATPD diagnoses in Commercial are unlikely to be affected by this difference in service configuration. Because of this case-mix, the incidence of psychosis was about half in Commercial (1.43/2.57 at two years, reflected by a lower mean value of the PI). The most important difference is that, while previous replications were performed in data collected from publicly funded secondary mental healthcare alone, the Commercial database was composed of both primary and secondary healthcare data composed of commercially insured patients. Given such relevant differences, it was expected that the risk calculator could not be easily transported to the Commercial setting and that it would achieve a lower prognostic performance and calibration than that observed in the first two external validations.

Despite these differences in clinical setting and populations, the overall prognostic accuracy of the transdiagnostic, clinically-based risk calculator remained significantly above chance. As expected, the level of prognostic performance (Harrell’s C = 0.68) was suboptimal and lower than our previous external validation (Harrell’s C = 0.73)^11^. Yet, this level of accuracy is comparable to that of structural neuroimaging methods (i.e., gray matter volume) to detect a first-episode of psychosis at the individual level, with accuracies ranging from 0.5 to 0.63^37^. A recent machine-learning study externally validated a risk calculator to predict treatment outcome in depression in 151 patients. The study reported a one year prognostic accuracy of 0.59 and concluded that, if implemented at scale, performance even only significantly above chance can be considered to be clinically useful^38^. Given that our risk calculator has been developed on real-world EHR data, it offers the potential for automatically screening large mental health populations. Psychiatry is undergoing a digital revolution^39^, and there is an ongoing expansion of EHR adoption worldwide. More to this point, this risk calculator was evidently developed with a clear vision of future implementation as decision support in clinical routine and is currently being piloted in this capacity^12,15^. For example, it uses simple predictors that can easily be understood by clinicians, as compared to complex black-box machine-learning-derived algorithms^40^. Furthermore, harnessing data from EHRs is cheaper than other methods such as patient recruitment, because most of the predictors are available as part of clinical routine. There are no competing algorithms (CHR-P instruments are not usable for screening purposes)^41^ to screen the at-risk population at scale. Other risk prediction tools in early psychosis have shown promise, however they predominantly rely on clinical symptom scores^42,43^, which means they are more financially and labor intensive than this tool; potential for automation is therefore limited. Moreover, these tools are focused on identifying transition to psychosis and are reliant on prior identification of CHR-P, whereas our tool is able to predict psychosis risk transdiagnostically outside of this designation. Thus, there is potential benefit in utilizing this risk calculator to screen for psychosis risk in large numbers.

There is scope for optimization of the current risk calculator through stepped risk stratification and model refinement. As a first step, this risk calculator could be deployed in a screening pathway where an individual’s risk is calculated upon entry into secondary mental health services. Individuals flagged by our risk calculator as being at risk for psychosis would progress to a more thorough clinical CHR-P assessment in the context of a staged sequential risk assessment^44,45^. This could supplement other detection strategies targeting the general population, such as the Youth-Mental Risk and Resilience study (YouR-Study)^46^, which provided the first evidence of digital detection tools improving identification of psychosis in the general population. A potential further step would be combining the risk calculator with additional information (environmental, genetic or biomarkers) to improve prognostic accuracy further^44,45,47^, refine estimates of individuals’ risk and stratify them accordingly. This is in keeping with the current clinical staging model of early psychosis, which aims to improve preventative care and reduce the duration of untreated psychosis to improve outcomes^1^. In addition to its clinical utility, this risk calculator could improve CHR-P research by aiding recruitment for much needed large-scale international collaborations in the vein of the HARMONY project, incorporating NAPLS (https://campuspress.yale.edu/napls/), PRONIA (https://www.pronia.eu/) and PSYSCAN (http://psyscan.eu), and the proposed 26-site ProNET cohort study. Furthermore, this prognostic model can be refined. In companion studies, we have tested whether using machine-learning methods and expanding the range of^48^, or redefining^49^, predictors might improve the prognostic accuracy of this risk calculator.

The limitations of this study are largely inherited from the original study. We did not employ structured psychometric interviews to ascertain the type of emerging psychotic diagnoses at follow-up. However, we predicted psychotic disorders rather than specific ICD-10 diagnoses, a category which has good prognostic stability^50^. Therefore, while the psychotic diagnoses in our analyses are high in ecological validity (i.e., they represent real-world clinical practice), they have not been subjected to formal validation with research-based criteria. However, the use of structured diagnostic interviews can lead to selection biases, decreasing the transportability of models^51^. There is also meta-analytical evidence indicating that within psychotic disorders, administrative data recorded in clinical registers are generally predictive of true validated diagnoses^52^.

Other limitations were inherent in the Commercial database, mostly due to the lack of patient-level ethnicity data and a short follow-up time. These two issues reduced the prognostic performance of the model a priori, in particular considering that risk for psychosis may well extend beyond two years^53^. It is therefore possible that prognostic performance of this model in the longer term may actually be better than the performance reported here. A further limitation is that the study team for this replication is not completely independent from the team who completed the original study^54^, which is particularly relevant given the support of a pharmaceutical company. However, Lundbeck has no financial interests nor patents on this project. As this study involved a large commercial dataset and a refined version of the model, it was logistically impossible to conduct this research independently from the original team. To mitigate against this overlap, we adhered to the Royston^22^, RECORD^13^, and TRIPOD^19^ guidelines to ensure transparency. Finally, although we welcome further external validation studies, it must be noted that even strong replication does not automatically imply the potential for successful adoption in clinical or public health practice. Ideally, randomized clinical trials or economic modeling are needed to assess whether our risk calculator effectively improves patient outcomes.

## Conclusion

The largest international external replication of an individualized prognostic model in psychiatry confirms that precision medicine in this discipline is feasible even at large scale. The transdiagnostic, individualized, clinically-based risk calculator is potentially transportable on an international scale to improve the automatic detection of individuals at risk of psychosis. Further research should refine the model and test the benefit of implementing this risk prediction model in clinical routine.

## Supplementary information

Supplementary
