# Supplementary material for: Transdiagnostic individualized clinically-based risk calculator for the automatic detection of individuals at-risk and the prediction of psychosis: external replication in 2,430,333 US patients
Source: Transl Psychiatry. 2020 Oct 29;10:364. doi: 10.1038/s41398-020-01032-9 (PMC7596040; doi:10.1038/s41398-020-01032-9)
Supplement: Supplementary file 1 — Supplementary [file 41398_2020_1032_MOESM1_ESM.docx]

**Supplementary Online Content**

Oliver D, Wong J, Bøg M et al. Transdiagnostic Individualized Clinically-based Risk Calculator for the Automatic Detection of Individuals at-Risk and the Prediction of Psychosis: External Replication in 2,430,333 US Patients

**Table S1.** REporting of studies Conducted using Observational Routinely-collected Data (RECORD) statement

**Methods S1.** Description of IBM^®^ MarketScan^®^ Commercial Database

**Methods S2.** Definition of emerging ICD-10 primary diagnosis of non-organic psychotic disorder

**Table S2.** Predictor definitions: Primary index diagnoses of non-organic and non-psychotic mental disorder formulated at baseline (time of the first contact with the NHS Trust).

**Table S3.** Predictor definitions: self-assigned ethnicity

**Methods S3.** Definition of lookback period

**Table S4.** Model performance of the Transdiagnostic Individualised Clinically-based Risk Calculator for the Automatic Detection of Individuals at Risk and the Prediction of Psychosis (revised version) in the original South London and Maudsley NHS Foundation Trust (SLaM) derivation and validation datasets.

**Table S5.** Transdiagnostic Individualised Clinically-based Risk Calculator for the Automatic Detection of Individuals at Risk and the Prediction of Psychosis (revised version), original derivation dataset (SLaM boroughs Lambeth & Southwark). This model has been used for external validation in the current study.

**Table S6.** Model performance of the Transdiagnostic Individualised Clinically-based Risk Calculator for the Automatic Detection of Individuals at Risk and the Prediction of Psychosis (revised version) in the original SLaM derivation dataset with removed ethnicity predictor and with aggregate ethnicity

coefficients.

**References**

**Table S1.** The REporting of studies Conducted using Observational Routinely-collected Data (RECORD) statement – checklist of items, extended from the STrengthening the Reporting of OBservational studies in Epidemiology (STROBE) statement, that should be reported in observational studies using routinely collected health data.

|  | **Item no.** | **STROBE items** | **Location in manuscript where items are reported** | **RECORD items** | **Location in manuscript where items are reported** |
| --- | --- | --- | --- | --- | --- |
| **Title and abstract** | | | | | |
|  | 1 | (a) Indicate the study’s design with a commonly used term in the title or the abstract (b) Provide in the abstract an informative and balanced summary of what was done and what was found. | Abstract | RECORD 1.1: The type of data used should be specified in the title or abstract. When possible, the name of the databases used should be included.  RECORD 1.2: If applicable, the geographic region and timeframe within which the study took place should be reported in the title or abstract.  RECORD 1.3: If linkage between databases was conducted for the study, this should be clearly stated in the title or abstract. | Abstract  Abstract  NA |
| **Introduction** | | | | | |
| Background rationale | 2 | Explain the scientific background and rationale for the investigation being reported. | Introduction |  |  |
| Objectives | 3 | State specific objectives, including any prespecified hypotheses. | Abstract, Introduction, Materials and methods |  |  |
| **Methods** | | | | | |
| Study Design | 4 | Present key elements of study design early in the paper. | Abstract |  |  |
| Setting | 5 | Describe the setting, locations, and relevant dates, including periods of recruitment, exposure, follow-up, and data collection. | Abstract, Materials and methods |  |  |
| Participants | 6 | *(a) Cohort study* - Give the eligibility criteria, and the sources and methods of selection of participants. Describe methods of follow-up  *Case-control study* - Give the eligibility criteria, and the sources and methods of case ascertainment and control selection. Give the rationale for the choice of cases and controls  *Cross-sectional study* - Give the eligibility criteria, and the sources and methods of selection of participants  *(b) Cohort study* - For matched studies, give matching criteria and number of exposed and unexposed  *Case-control study* - For matched studies, give matching criteria and the number of controls per case | Abstract  Materials and methods  NA  NA  NA  NA | RECORD 6.1: The methods of study population selection (such as codes or algorithms used to identify subjects) should be listed in detail. If this is not possible, an explanation should be provided.  RECORD 6.2: Any validation studies of the codes or algorithms used to select the population should be referenced. If validation was conducted for this study and not published elsewhere, detailed methods and results should be provided.  RECORD 6.3: If the study involved linkage of databases, consider use of a flow diagram or other graphical display to demonstrate the data linkage process, including the number of individuals with linked data at each stage. | Materials and methods  Materials and methods, referenced previous publications addressing the codes or algorithms used  NA |
| Variables | 7 | Clearly define all outcomes, exposures, predictors, potential confounders, and effect modifiers. Give diagnostic criteria, if applicable. | Materials and methods, Table 1 | RECORD 7.1: A complete list of codes and algorithms used to classify exposures, outcomes, confounders, and effect modifiers should be provided. If these cannot be reported, an explanation should be provided. | Materials and methods, Methods 1, Methods 2, Table 5 |
| Data sources/ measurement | 8 | For each variable of interest, give sources of data and details of methods of assessment (measurement).  Describe comparability of assessment methods if there is more than one group. | Materials and methods |  |  |
| Bias | 9 | Describe any efforts to address potential sources of bias. | Materials and methods |  |  |
| Study size | 10 | Explain how the study size was arrived at. | Materials and methods  Figure 1 |  |  |
| Quantitative variables | 11 | Explain how quantitative variables were handled in the analyses. If applicable, describe which groupings were chosen, and why. | Statistical analysis |  |  |
| Statistical methods | 12 | (a) Describe all statistical methods, including those used to control for confounding  (b) Describe any methods used to examine subgroups and interactions  (c) Explain how missing data were addressed  (d) *Cohort study* - If applicable, explain how loss to follow-up was addressed  *Case-control study* - If applicable, explain how matching of cases and controls was addressed  *Cross-sectional study* - If applicable, describe analytical methods taking account of sampling strategy  (e) Describe any sensitivity analyses | Statistical analysis  Statistical analysis  Statistical analysis  Statistical analysis  NA  NA  NA |  |  |
| Data access and cleaning methods |  | .. |  | RECORD 12.1: Authors should describe the extent to which the investigators had access to the database population used to create the study population.  RECORD 12.2: Authors should provide information on the data cleaning methods used in the study. | Discussion  NA |
| Linkage |  | .. |  | RECORD 12.3: State whether the study included person-level, institutional-level, or other data linkage across two or more databases. The methods of linkage and methods of linkage quality evaluation should be provided. | NA |
| **Results** | | | | | |
| Participants | 13 | (a) Report the numbers of individuals at each stage of the study (*e.g.*, numbers potentially eligible, examined for eligibility, confirmed eligible, included in the study, completing follow-up, and analysed)  (b) Give reasons for non-participation at each stage.  (c) Consider use of a flow diagram. | Results  NA  Figure 1 | RECORD 13.1: Describe in detail the selection of the persons included in the study (*i.e.,* study population selection) including filtering based on data quality, data availability and linkage. The selection of included persons can be described in the text and/or by means of the study flow diagram. | Results |
| Descriptive data | 14 | (a) Give characteristics of study participants (*e.g.*, demographic, clinical, social) and information on exposures and potential confounders  (b) Indicate the number of participants with missing data for each variable of interest  (c) *Cohort study* - summarise follow-up time (*e.g.*, average and total amount). | Table 1  Figure 1  Results |  |  |
| Outcome data | 15 | *Cohort study* - Report numbers of outcome events or summary measures over time  *Case-control study* - Report numbers in each exposure category, or summary measures of exposure  *Cross-sectional study* - Report numbers of outcome events or summary measures | Results and Figure 2  NA  NA |  |  |
| Main results | 16 | (a) Give unadjusted estimates and, if applicable, confounder-adjusted estimates and their precision (e.g., 95% confidence interval). Make clear which confounders were adjusted for and why they were included  (b) Report category boundaries when continuous variables were categorized  (c) If relevant, consider translating estimates of relative risk into absolute risk for a meaningful time period. | Results  NA  Figure 2 |  |  |
| Other analyses | 17 | Report other analyses done—e.g., analyses of subgroups and interactions, and sensitivity analyses. | Results |  |  |
| **Discussion** | | | | | |
| Key results | 18 | Summarise key results with reference to study objectives. | First paragraph of discussion |  |  |
| Limitations | 19 | Discuss limitations of the study, taking into account sources of potential bias or imprecision. Discuss both direction and magnitude of any potential bias. | Discussion (strengths and weaknesses of the study section) | RECORD 19.1: Discuss the implications of using data that were not created or collected to answer the specific research question(s). Include discussion of misclassification bias, unmeasured confounding, missing data, and changing eligibility over time, as they pertain to the study being reported. | Discussion (implications for clinical practice and strengths and weaknesses of the study sections) |
| Interpretation | 20 | Give a cautious overall interpretation of results considering objectives, limitations, multiplicity of analyses, results from similar studies, and other relevant evidence. | Discussion (interpretation of findings and strengths and weaknesses of the study sections) |  |  |
| Generalisability | 21 | Discuss the generalisability (external validity) of the study results. | Discussion |  |  |
| **Other information** | | | | | |
| Funding | 22 | Give the source of funding and the role of the funders for the present study and, if applicable, for the original study on which the present article is based. | Financial support statement in the abstract |  |  |
| Accessibility of protocol, raw data, and programming code |  | .. |  | RECORD 22.1: Authors should provide information on how to access any supplemental information such as the study protocol, raw data, or programming code. | Statistical analysis, Methods 2 |

**Methods S1.** Description of IBM^®^ MarketScan^®^ Commercial Database

This database is based on data from Truven MarketScan Commercial Claims and Encounters Database (“Commercial database” in short), which contains information from active employees, early retirees, Consolidated Omnibus Budget Reconciliation Act continues, and dependents insured by employer-sponsored plans (i.e. individuals not eligible for Medicare). It incorporates data from hundreds of payers, including commercial insurance companies, Blue Cross Blue Shield plans, and third-party administrators. It incorporates data from hundreds of payers, including commercial insurance companies and third-party administrators.

**Methods S2.** Definition of emerging ICD-10 primary diagnosis of non-organic psychotic disorder

This was defined as the emergence of the first ICD-10 primary diagnosis of non-organic psychotic disorder after the index diagnosis as recorded in the local electronic medical records: schizophrenia spectrum psychoses (schizophrenia [F20.x, except F20.4/F20.5], schizoaffective disorder [F25.x], delusional disorders [F22.x, F24], Acute and Transient Psychotic Disorders [ATPD, F23.x]), unspecified nonorganic psychosis (F28/F29), psychotic disorders due to psychoactive substance use ([F10-F19].5), and affective psychoses (mania with psychotic symptoms [F30.2], bipolar affective disorder with psychotic symptoms [F31.2, F31.5], and depression with psychotic symptoms [F32.3/F33.3]). Accordingly, baseline ICD-10 psychotic disorders were excluded, with the exception of ATPD (F23.x), which are, by definition, clinically remitting and non-psychotic within three months (short-lived). The rationale for including the ATPD is due to the fact that this group is prognostically similar to the Brief Limited Intermittent Psychotic Symptom (BLIPS) or Brief Intermittent Psychotic Symptoms (BIPS) subgroups of the CHR-P construct (for details on these competing operationalisation see previous publications on the diagnostic and prognostic significance of BLIPS^1,2^). On a diagnostic level, about two thirds (68%) of BLIPS meet ATPD criteria^1^.

**Table S2**. Predictor definitions: Primary index diagnoses of non-organic and non-psychotic mental disorder formulated at baseline (time of the first contact with the NHS Trust).

| **Primary index diagnosis** | **ICD-10 code** | **ICD-10 diagnosis name** |
| --- | --- | --- |
| Acute and transient psychotic disorders | F23.x | Acute and transient psychotic disorders |
| Substance use disorders | F10 (excluding F10.4, F10.5, F10.7) | Non-psychotic mental and behavioural disorders due to use of alcohol |
|  | F11 (excluding F11.4, F11.5, F11.7) | Non-psychotic mental and behavioural disorders due to use of opioids |
|  | F12 (excluding F12.4, F12.5, F12.7) | Non-psychotic mental and behavioural disorders due to use of cannabinoids |
|  | F13 (excluding F13.4, F13.5, F13.7) | Non-psychotic mental and behavioural disorders due to use of sedatives or hypnotics |
|  | F14 (excluding F14.4, F14.5, F14.7) | Non-psychotic mental and behavioural disorders due to use of cocaine |
|  | F15 (excluding F15.4, F15.5, F15.7) | Non-psychotic mental and behavioural disorders due to use of other stimulants, including caffeine |
|  | F16 (excluding F16.4, F16.5, F16.7) | Non-psychotic mental and behavioural disorders due to use of hallucinogens |
|  | F17 (excluding F17.4, F17.5, F17.7) | Non-psychotic mental and behavioural disorders due to use of tobacco |
|  | F18 (excluding F18.4, F18.5, F18.7) | Non-psychotic mental and behavioural disorders due to use of volatile solvents |
|  | F19 (excluding F19.4, F19.5, F19.7) | Non-psychotic mental and behavioural disorders due to multiple drug use and use of other psychoactive substances |
| Bipolar mood disorders | F31.x (excluding F31.2 and F31.5) | Non-psychotic bipolar disorder |
|  | F34.0 | Cyclothymia |
|  | F30.x (excluding F30.2) | Non-psychotic mania or hypomania |
| Non-bipolar mood disorders | [F32-F33].x (excluding F32.3 and F33.3) | Non-psychotic depressive disorder |
|  | F34.1 | Dysthymia |
|  | F34.8, F34.9, F38.x, F39 | Unspecified mood disorders |
| Anxiety disorders | F40.x | Phobic anxiety disorders |
|  | F41.0 | Panic disorder |
|  | F41.1 | Generalised anxiety disorder |
|  | F41.2-F41.9 | Other anxiety disorders |
|  | F42.x | Obsessive compulsive disorders |
|  | F43.x | Reaction to severe stress, and adjustment disorders |
|  | F44.x | Dissociative [conversion] disorders |
|  | F45.x | Somatoform disorders |
|  | F48.x | Other neurotic disorders |
| Personality disorders | F60.0 | Paranoid personality disorder |
|  | F60.1 | Schizoid personality disorder |
|  | F60.2 | Dissocial personality disorder |
|  | F60.3 | Emotionally unstable personality disorder |
|  | F60.4 | Histrionic personality disorder |
|  | F60.5 | Anankastic personality disorder |
|  | F60.6 | Anxious [avoidant] personality disorder |
|  | F60.7 | Dependent personality disorder |
|  | F60.8-F60.9, F61, F62.x, F68.x, F69 | Other personality disorders |
|  | F21 | Schizotypal Disorder |
|  | F63.x | Habit and impulse disorders |
|  | F64.x, F65.x, F66.x | Sexual disorders |
| Developmental disorders | F80.x | Specific developmental disorders of speech and language |
|  | F81.x, F82, F83 | Other specific developmental disorders |
|  | F84.x | Pervasive developmental disorders |
|  | F88, F89 | Other and unspecified disorders of psychological development |
| Childhood/adolescence onset disorders | F90.x | Hyperkinetic disorders |
|  | F91.x | Conduct disorders |
|  | F92.x, F93.x, F94.x, F98.x | Other emotional and behavioural disorders with childhood or adolescence onset |
|  | F95.x | Tic disorders |
| Physiological syndromes | F50.x | Eating disorders |
|  | F51.x | Nonorganic sleep disorders |
|  | F52.x | Sexual dysfunction, not caused by organic disorder or disease |
|  | F53.x (excluding F53.1) | Non-psychotic Mental and behavioural disorders associated with the puerperium, not elsewhere classified |
|  | F54.x, F55, F59 | Other physiological syndromes |
| Mental retardation | F70.x | Mild mental retardation |
|  | F71.x | Moderate mental retardation |
|  | F72.x | Severe mental retardation |
|  | F73.x | Profound mental retardation |
|  | F78.x | Other mental retardation |
|  | F79.x | Unspecified mental retardation |
| *F00-F09 organic mental disorders and all psychotic disorders other than F23.x were excluded* | | |

| **Table S3**. Predictor definitions: self-assigned ethnicity. Individual-level ethnicity data was not available for the MarketScan database so was imputed using Integrated Public Use Microdata Series (IPUMS) as discussed in the methods section | | |  |
| --- | --- | --- | --- |
| **Ethnic group** | **Ethnicity as recorded in patient electronic health records** | **Ethnicity as recorded in IPUMS** | |
| Black | Black or Black British - African  Black or Black British - Caribbean  Black or Black British - Any other Black background | prop_Black_African_American | |
| White | White - British  White - Irish  White - Any other White background | prop_White | |
| Asian | Asian or Asian British - Bangladeshi  Asian or Asian British - Indian  Asian or Asian British - Pakistani  Asian or Asian British - Any other Asian background  Other Ethnic Groups - Chinese | prop_Japanese  prop_Chinese  prop_Other_Asian_or_Pacific_Isla | |
| Mixed | Mixed - White and Asian  Mixed - White and Black African  Mixed - White and Black Caribbean  Mixed - Any other mixed background | prop_Two_major_races  prop_Three_or_more_major_races | |
| Other | Other Ethnic Groups - Any other ethnic group | prop_American_Indian_or_Alaska_N  prop_Other_race__nec | |
| Missing | Not Known  Not Recorded |  | |

**Methods S3.** Definition of lookback period

A look back period is the length of an individual’s medical history prior to index diagnosis. This means that additional diagnoses, potentially unreported at index, are more likely to be detected. In particular, this limits discharge coding bias, where less serious, chronic conditions may be unrecorded within Electronic Health Records (EHRs) when a patient is being treated for an acute episode, and, likewise, if acute episodes are missed due to ongoing chronic symptomology. As such, a look back period of six months was applied to each patient to ensure stability of diagnoses

**Table S4.** Model performance of the Transdiagnostic Individualised Clinically-based Risk Calculator for the Automatic Detection of Individuals at Risk and the Prediction of Psychosis (revised version) in the original South London and Maudsley NHS Foundation Trust (SLaM) derivation and validation datasets.

|  | | | | | | | | |  |
| --- | --- | --- | --- | --- | --- | --- | --- | --- | --- |
| Performance measure | | Derivation (n = 34,209) ^(a)^ | | |  | Validation (n = 54,716) ^(b)^ | | | |
|  | | Mean 95%CI | | |  | Mean 95%CI | | | |
| *Overall* | |  |  |  |  |  |  |  | |
|  | Brier ^(c)^ | 0.028 |  |  |  | 0.021 |  |  | |
|  | R^2^ | 0.746 | 0.704 | 0.785 |  | 0.719 | 0.673 | 0.761 | |
| *Discrimination* | |  |  |  |  |  |  |  | |
|  | Harrell's C | 0.809 | 0.795 | 0.822 |  | 0.790 | 0.775 | 0.806 | |
| *Calibration* | |  |  |  |  |  |  |  | |
|  | Calibration slope | 1 |  |  |  | 0.968 | 0.929 | 1.015 | |
| a) The sample size is larger than the original 33,820 because individuals with psychosis onset within the first 3 months since baseline were not excluded.  b) The sample size of the external validation matches that used in the original study to facilitate comparability.  c) at 10-years. | | | | | | | | |  |

**Table S5.** Transdiagnostic Individualised Clinically-based Risk Calculator for the Automatic Detection of Individuals at Risk and the Prediction of Psychosis (revised version), original derivation dataset (SLaM boroughs Lambeth & Southwark). This model has been used for external validation in the current study.

|  |  | Multivariable model | | | |  |
| --- | --- | --- | --- | --- | --- | --- |
| *Predictor* | | *Beta coefficient* | *95% CI* | | *P* |  |
| Age (years) | | 0.010 | 0.005 | 0.143 | <0.001 |  |
| Gender | |  |  |  |  |  |
|  | Male | 0.457 | 0.185 | 0.730 | 0.001 |  |
|  | Female (R) | 0 |  |  |  |  |
| Age by gender (male) | | -0.009 | -0.015 | -0.002 | 0.009 |  |
| Ethnicity | |  |  |  |  |  |
|  | White (R) | 0 |  |  |  |  |
|  | Black | 0.995 | 0.865 | 1.125 | <0.001 |  |
|  | Asian | 0.487 | 0.207 | 0.767 | 0.001 |  |
|  | Mixed | 0.686 | 0.372 | 1.000 | <0.001 |  |
|  | Other | 0.340 | 0.143 | 0.537 | 0.001 |  |
| ICD-10 diagnostic spectra | |  |  |  |  |  |
|  | CHR-P (R) | 0 |  |  |  |  |
|  | Acute and transient psychotic disorders | 1.169 | 0.876 | 1.464 | <0.001 |  |
|  | Substance use disorders | -1.748 | -2.065 | -1.431 | <0.001 |  |
|  | Bipolar mood disorders | 0.003 | -0.327 | 0.333 | 0.986 |  |
|  | Non-bipolar mood disorders | -1.560 | -1.876 | -1.245 | <0.001 |  |
|  | Anxiety disorders | -2.006 | -2.320 | -1.691 | <0.001 |  |
|  | Personality disorders | -1.363 | -1.754 | -0.971 | <0.001 |  |
|  | Developmental disorders | -3.337 | -4.018 | -2.656 | <0.001 |  |
|  | Childhood/adolescence onset disorders | -3.200 | -3.641 | -2.753 | <0.001 |  |
|  | Physiological syndromes | -2.310 | -2.764 | -1.856 | <0.001 |  |
|  | Mental retardation | -2.326 | -2.864 | -1.788 | <0.001 |  |
| (R) Reference category | | | | | |  |
|  | | | | | | |
|  |  |  |  |  |  |  |

**Table S6.** Model performance of the Transdiagnostic Individualised Clinically-based Risk Calculator for the Automatic Detection of Individuals at Risk and the Prediction of Psychosis (revised version) in the original SLaM derivation dataset with removed ethnicity predictor and with aggregate ethnicity coefficients.

| Performance measure | | |  | | |  |
| --- | --- | --- | --- | --- | --- | --- |
|  | | | Mean 95%CI | | |  |
| *No ethnicity* | | |  |  |  |  |
|  | Harrell's C (mean, 95%CI) | | 0.790 | 0.776 | 0.803 |  |
| *Aggregate ethnicity coefficients* | |  |  |  |  |  |
|  | Harrell's C (mean, 95%CI) | | 0.761 | 0.745 | 0.776 |  |

**References**

1 Fusar-Poli P, Cappucciati M, De Micheli A, Rutigliano G, Bonoldi I, Tognin S *et al.* Diagnostic and prognostic significance of brief limited intermittent psychotic symptoms (BLIPS) in individuals at ultra high risk. *Schizophr Bull* 2017; **43**: 48–56.

2 Fusar-Poli P, Cappucciati M, Bonoldi I, Hui LMC, Rutigliano G, Stahl DR *et al.* Prognosis of Brief Psychotic Episodes: A Meta-analysis. *JAMA Psychiatry* 2016; **73**: 211–220.
